# Ocular Side Effects of EGFR-Inhibitor ABT-414 in Recurrent Glioblastoma: A Long-Term Safety Study

**DOI:** 10.3389/fonc.2020.593461

**Published:** 2020-10-14

**Authors:** Raffaele Parrozzani, Giuseppe Lombardi, Edoardo Midena, Davide Londei, Marta Padovan, Giulia Marchione, Mario Caccese, Giulia Midena, Vittorina Zagonel, Luisa Frizziero

**Affiliations:** ^1^Department of Ophthalmology, University of Padova, Padova, Italy; ^2^Department of Oncology, Oncology 1, Veneto Institute of Oncology-IRCCS, Padua, Italy; ^3^IRCCS—Fondazione Bietti, Rome, Italy; ^4^Unità Operativa Complessa Oftalmologia, Fondazione Policlinico Universitario A. Gemelli IRCCS, Rome, Italy

**Keywords:** ABT-414, confocal microscopy, cornea, epidermal growth factor receptor-inhibitor, glioblastoma, side effects, sub-basal nerve plexus, long-term follow-up

## Abstract

This study aimed to prospectively evaluate, on a long-term basis, corneal side effects secondary to compassionate administration of epidermal growth factor receptor (EGFR) inhibitor depatuxizumab mafodotin (ABT-414) in patients affected by EGFR-amplified recurrent glioblastoma. Fifteen patients with a median follow-up of 4.3 months after treatment discontinuation were enrolled. Each patient underwent full ophthalmologic examination including *in vivo* corneal confocal microscopy (CCM). No CTCAE grade 4 toxicity and four (27%) grade 3 toxicities were documented during treatment. Ocular symptoms (blurred vision, eye pain, photophobia) were experienced by all patients, reaching maximal severity after the second ABT-414 infusion, with persistence until treatment discontinuation. During treatment, CCM documented specific changes in the corneal epithelium and in the sub-basal nerve plexus layer fibers of all eyes. The median time of symptoms resolution after treatment discontinuation ranged from 38 days (eye pain) to 53 days (photophobia). The median time of signs resolution ranges from 14 days (corneal ulcer) to 38 days (superficial punctate epitheliopathy, corneal stroma edema and intraepithelial cysts). ABT-414 corneal side effects are detectable in all treated patients. Related symptoms are gradually experienced by all patients during treatment and although reversible, they are characterized by a relative prolonged persistence after treatment discontinuation.

## Introduction

Glioblastoma (GBM) is the most common primary malignant tumor of the central nervous system in adults, accounting for almost 50% of all malignant primary tumors of the brain, with a 5-years overall survival of 5% (1). Approximately 50% of GBMs harbor epidermal growth factor receptor (EGFR) gene amplification, causing the consequent overexpression of the EGFR protein (2, 3). EGFR-inhibitors are commonly used in the treatment of different EGFR amplified cancers, including colorectal, lung, breast, pancreatic, renal, head and neck, gynecologic, prostate and central nervous system tumors (2–6). These drugs have been proved to improve the prognosis of patients affected these tumors, suggesting a possible use in patients affected by EGFR amplified GBM (1–6).

Antibody-drug conjugates (ADC), a class of anticancer drugs, are composed by monoclonal antibodies with specific targeting properties and cytotoxic agents characterized by anti-tumor effects. Depatuxizumab mafodotin (Depatux-m), formerly ABT-414, is a newer generation ADC consisting of a veneered humanized recombinant immunoglobulin G1κ antibody that has binding properties specific for a unique epitope of human EGFR, which is attached with non-cleavable maleimido-caproyl linkers to a potent anti-microtubule agent, monomethylauristatin-F (MMAF) (2, 3, 7, 8). The recent phase-II controlled study on ABT-414 in the treatment of EGFR amplified recurrent GBM (NTELLANCE 2; NCT02343406) has suggested a possible role of this agent (in combination with temozolomide) in the treatment of this tumor. Unfortunately, the ocular toxicity related to the attached toxin has interfered with ABT-414 dose intensity, probably affecting also the treatment outcome of the clinical trial (3).

Cornea and conjunctiva are characterized by the constitutive localization of EGFR in the basal epithelial cells, and corneal toxicity is considered the most frequent side effects in patients treated by monoclonal antibodies against EGFR (2, 3, 9–14). In a recent study, our group has prospectively analyzed ABT-414 ocular side effects in a smaller cohort of patients affected by EGFR-amplified recurrent GBM using in-vivo clinical confocal microscopy (CCM), demonstrating that ABT-414 toxicity is not only directed to the corneal epithelium, but also to corneal nerves, and that corneal side effects are detectable in all treated patients (15). This study also suggested that ocular side effects improve upon treatment discontinuation. However, a precise definition of a median time to resolution was not possible because of follow-up loss for progressive disease in most of the patients. The aim of the present study is to further evaluate corneal side effects secondary to compassionate administration of EGFR inhibitor ABT-414 using CCM in a larger number of patients with a longer follow-up, and to obtain a mean time to resolution of these side effects.

## Materials and Methods

This was an institutional, observational, case-series with prospective enrollment, compliant with the tenets of the Declaration of Helsinki and approved by the Institutional Review Board (IRB) of the Veneto Institute of Oncology IOV-IRCCS (No. 08.2019). Patients affected by EGFR-amplified recurrent GBM planned to be treated with ABT-414 (compassionate use) plus temozolomide were consecutively recruited. Informed consent was obtained from each patient. Treatment with ABT-414 (1.5 mg/kg) every 14 day plus temozolomide 150–200 mg/m2/die for 5 consecutive days every 28 days was continued until either intolerable toxicity or disease progression, as assessed using response assessment in neuro-oncology (RANO) criteria (16). Each enrolled subject underwent full ophthalmologic examination at baseline and every 14 days during treatment. Follow-up after treatment discontinuation was performed at 1 month and every 2 months thereafter, as long as patient conditions allowed it. Adjunctive clinical examinations were eventually planned according to clinical indication. BCVA was measured using the ETDRS protocol (17). Slit lamp examination and fluorescein and lissamine green corneal staining were also performed. The presence of ocular symptoms and signs was graded using the CTCAE Version 4.0 (**Table 1**). The superficial punctate epitheliopathy was graded using the Oxford grading scheme (18). *In vivo* CCM of the cornea was performed at baseline and during follow-up using Heidelberg Retina Tomography with the Rostock Cornea Module (HRTIII/RCM, Heidelberg Engineering, Germany), as previously reported (15). The presence of multiple and diffuse epithelial hyperreflective white round spots and the presence of round cystic structures in the corneal epithelium were separately graded as mild (≤ 5 in a single CCM image) moderate (5 to 10 in a single CCM image) and severe (≥ 10). The presence of sub-basal plexus fibers toxicity was also graded as moderate (fragmentation and reduction of sub-basal plexus fibers compared to baseline) and severe (complete or almost complete disappearance of the sub-basal plexus fibers). All patients receive a standard topical therapy since treatment initiation (Hyaluronic acid 0.2% eyedrop four time a day). Patients developing corneal ulcer were treated by topical antibiotics and steroids.

**Table 1:** Common Terminology Criteria for Adverse Events (CTCAE): examined eye disorder.

Grade					
Adverse Event	1	2	3	4	5
Blurred vision	Intervention not indicated	Symptomatic; limiting instrumental ADL	Limiting self-care ADL	–	–
Conjunctivitis	Asymptomatic or mild symptoms; intervention not indicated	Symptomatic; topical Limiting self-care ADL intervention indicated (e.g., antibiotics)	Limiting self-care ADL	–	–
Corneal ulcer	–	Symptomatic; medical intervention indicated (e.g., topical agents); limiting instrumental ADL	Limiting self-care ADL; declining vision (worse than 20/40 but better than 20/200)	Perforation or blindness - (20/200 or worse) in the affected eye	–
Eye pain	Mild pain	Moderate pain; limiting instrumental ADL	Severe pain; limiting self-care ADL	–	–
Photophobia	Symptomatic but not limiting ADL	Limiting instrumental ADL	Limiting self-care ADL	–	–
Keratitis	–	Symptomatic; medical intervention indicated (e.g., topical agents) limiting instrumental ADL	Decline in vision (worse than 20/40 but better than 20/200); limiting self-care ADL	Perforation or blindness (20/200 or worse) in the affected eye	–

ADL, Activity of Daily Life.

## Results

### Baseline Characteristics

Fifteen patients affected by EGFR-amplified recurrent GBM were consecutively enrolled from January 2019 to May 2019 at Veneto Institute of Oncology-IRCCS, Padua, Italy. The median age at study inclusion was 57.2 years (range, 44.4–68.6). Enrolled patients undergone a median time of treatment of 2.3 months (95% CI 1.3–2.9), receiving a median of 4 infusions (range, 1–9), with a median of 3 ophthalmological evaluations during the active treatment phase (range, 2–4). Clinical and demographic characteristics of enrolled patients are reported in **Table 2**.

**Table 2:** Clinical and demographic characteristics.

Baseline characteristics	
No. of patients (eyes)	15 (30)
Gender (Male/Female)	9/6
	median (min – max)
Age (years) at study inclusion	57.2 (44.4–68.6)
Time (months) on ABT treatment	2.3 months (95% CI 1.3–2.9)
No. of ABT-414 cycles	4 (1–9)
No. of ophthalmological evaluations during treatments	3 (2–4)
Reasons for treatment discontinuation (permanently)	No (%)
-Disease progression	12 (80%)
-Withdrawal of consent	2 (13%)
- non-treatment-related cerebral hemorrhage	1 (7%)
Ophthalmological follow-up after drug discontinuation	
No. of evaluations, median (min-max)	7 (2–11)
Time (days) between each evaluation, median (min-max)	48 (10–70)
No. patients followed at least 30 days	15 (100%)
No. patients followed at least 90 days	7 (47%)
No. patients followed at least 150 days	6 (40%)
No. patients followed over 210 days	4 (27%)

No., Numbers; GBM, glioblastoma.

Five patients discontinued treatment temporarily and 2 patients reduced ABT dose to 1mg/Kg, mostly due to grade 2–3 ocular side effects. No patients permanently discontinued the treatment because of ocular toxicity. The reason of permanent treatment discontinuation was disease progression in twelve patients (80%), withdrawal of consent in two patients (13%), non-treatment-related cerebral hemorrhage (7%). After drug permanent discontinuation, all patients were followed for more than 30 days, 7 patients (47%) for more than 90 days, 6 patients (40%) for more than 150 days and 4 patients (27%) over 210 days. The median follow-up after treatment discontinuation was 4.3 months (range, 44–240 days; mean, 131 ± 74 days). The median numbers of ophthalmological evaluation after treatment discontinuation was 7 (range, 2–11), with a median interval between each evaluation of 48 days (range, 10–70). Mean best corrected visual acuity (BCVA) at baseline was 86 ± 3 Early Treatment Diabetic Retinopathy Study (ETDRS) score (**Table 3**) (17).

**Table 3:** Patients clinical characteristics during and after the treatment.

Patients characteristic	Baseline	At drug discontinuation	30 days	90 days	150 days	210 days	Mean time to resolution (days ± SD)	Median time to resolution (days)
**Patients in follow-up**	15(100%)	15 (100%)	15(100%)	8(47%)	6(40%)	4(27%)	N.A.	N.A.
**Symptoms^1^ No. of patients; (%) [grade of severity, median; max]^1^**								
Blurred vision	0 (0%) [N.A.]	15 (100%) [gr 1; gr 2]	11 (73%) [gr 2; gr2]	4 (57%) [gr 1; gr 2]	2 (33%) [gr 1; gr 1]	0 (0%) [N.A.]	66 ± 52	40
Eye pain	0 (0%) [N.A.]	12 (80%) [gr 2; gr 2]	8 (53%) [gr 1; gr 2]	3 (42%) [gr 1; gr 2]	1 (16%) [gr 1; gr 1]	0 (0%) [N.A.]	57 ± 43	38
Photophobia	0 (0%) [N.A.]	15 (100%) [gr 1; gr 2]	8 (53%) [gr 1; gr 2]	3 (42%) [gr 1; gr 2]	1 (16%) [gr 1; gr 1]	1 (25%) [gr 1; gr 1]	55 ± 53	53
**Signs^1^ No. of eye; (%) [grade of severity, median; max]^1^**								
Conjunctivitis	0 (0%) [N.A.]	30 (100%) [gr 2; gr 2]	24 (80%) [gr 1; gr 2]	6 (42%) [gr 1; gr 2]	2 (16%) [gr 1; gr 1]	1 (12%) [gr 1; gr 1]	58 ± 43	38
Corneal ulcer	0 (0%) [N.A.]	4 (13%)^2^ [gr 2; gr3]	2 (6%)^3^ [gr 2; gr2]	0 (0%) [N.A.]	0 (0%) [N.A.]	0 (0%) [N.A.]	23 ± 12	24
Keratitis	0 (0%) [N.A.]	30 (100%) [gr 2; gr 2]	24 (80%) [gr 1; gr 2]	6 (42%) [gr 1; gr 2]	2 (16%) [gr 1; gr 1]	1 (25%) [gr 1; gr 1]	58 ± 43	38
Conjunctival hyperemia	0 (0%)	28 (93%)	22 (73%)	6 (42%)	2 (16%)	1 (12%)	59 ± 45	37
Blepharitis	0 (0%)	14 (50%)	8 (47%)	2 (29%)	0 (0%)	0 (0%)	40 ± 29	37
Superficial punctate epitheliopathy	0 (0%)	30 (100%)	24 (80%)	4 (28%)	4 (33%)	2 (25%)	63 ± 53	38
Intraepithelial cysts	0 (0%)	30 (100%)	24 (80%)	6 (42%)	4 (33%)	0 (0%)	62 ± 45	38
Corneal stroma edema	0 (0%)	24 (80%)	22 (73%)	6 (42%)	2 (16%)	0 (0%)	62 ± 40	38
**Clinical laser scanning *in vivo* confocal microscopy (No. of eyes; %)**								
Basal epithelial hyperreflective spots	0 (0%)	30 (100%)	30 (100%)	6 (37%)	0 (0%)	0 (0%)	60 ± 32	40
Round cystic structure	0 (0%)	30 (100%)	30 (100%)	6 (37%)	4 (33%)	0 (0%)	66 ± 44	40
Fragmentation of sub-basal nerve plexus fibers	N.A.	30 (100%)	30 (100%)	6 (37%)	2 (16%)	0 (0%)	64 ± 40	40
**ETDRS score**	86±3 (79–88)	65±9 (53–74)	70±11 (57–78)	81± 9 (59–86)	83±4 (70–86)	84±4 (76–86)	63.1	

^1^Common Terminology Criteria for Adverse Events (CTCAE) Version 4.0; ^2^Four eyes of four different patients. ^3^Two eyes of two different patients; ETDRS, Early Treatment Diabetic Retinopathy Study; No., Number; N.A., not applicable; SD, Standard Deviation.

### Ocular Side Effects

The prevalence and severity (median and maximal severity) of ocular side effects are reported in **Table 3**. No Common Terminology Criteria for Adverse Events (CTCAE) grade 4 toxicity was documented. Four eyes of four patients (27%) developed grade 3 toxicity during treatment, after a median of 42 days and a median of 4 drug infusions. The presence of a grade 3 toxicity was related to the presence of a corneal ulcer in each case. These ulcers were characterized by a central location in all cases, a mean diameter of 3 mm (range, 1–6 mm), no relevant corneal thinning and a median time of resolution of 38 days. These patients were treated by topical topical antibiotics and steroids until resolution.

Symptoms were experienced gradually by all patients during treatment, starting a mean of 8 days after drug infusion (range, 5–20), and included: blurred vision (15 patients; 100%), eye pain (12 patients; 80%) and photophobia (15 patients; 100%) (**Table 3**). The maximal symptoms severity was reached after a median of 2 drug infusions (range, 2–4) and maintained in all cases until drug discontinuation.

**Figure 1:**
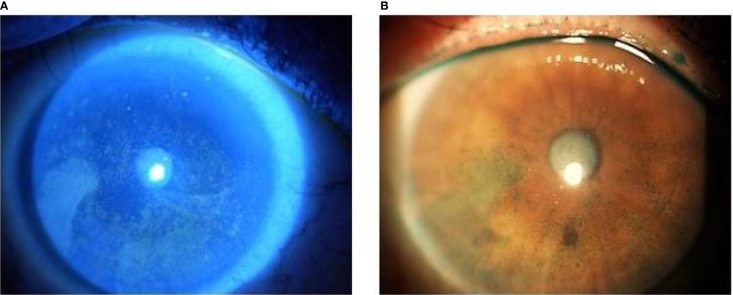
Biomicroscopy examination with **(A)** fluorescein and **(B)** lissamine green staining showing the corneal epithelial defects (yellow areas and green areas) secondary to ABT-414.

**Figure 2:**
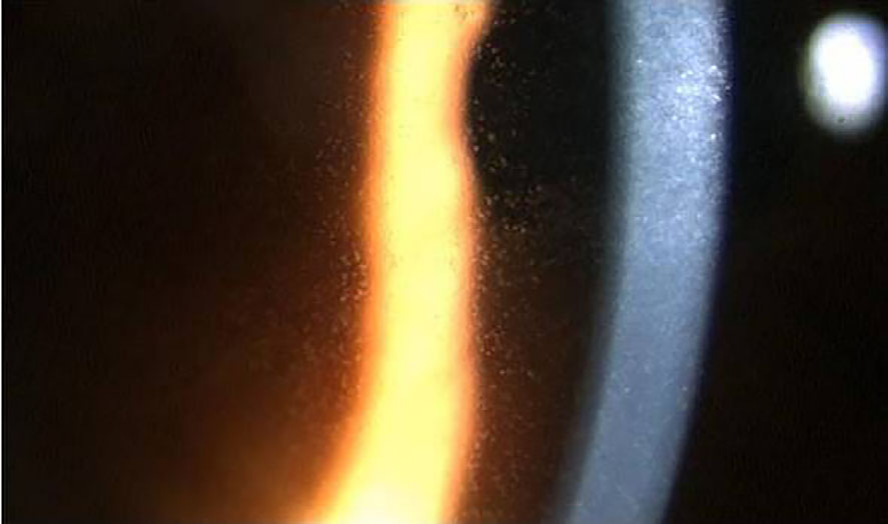
Biomicroscopy examination revealing the presence of corneal cysts secondary to ADT-414 toxicity.

The biomicroscopic examination documented the appearance of the typical ABT-414 corneal side effects, starting a mean of 8 days after the first drug infusion (range, 5–20), including: mild conjunctival hyperemia (28 eyes; 93%), mild blepharitis (14 eyes; 47%), superficial punctate epitheliopathy (30 eyes; 100%) (**Figure 1**) and multiple corneal epithelial cysts (30 eyes; 100%) (**Figure 2**).

The maximal severity of the superficial punctate epitheliopathy was graded as moderate in 16 cases and marked in other 14 cases, according to the Oxford grading (18). The maximal severity of these side effects was reached after the second ABT-414 infusion (range, 2–4) and maintained during the active treatment period.

**Figure 3:**
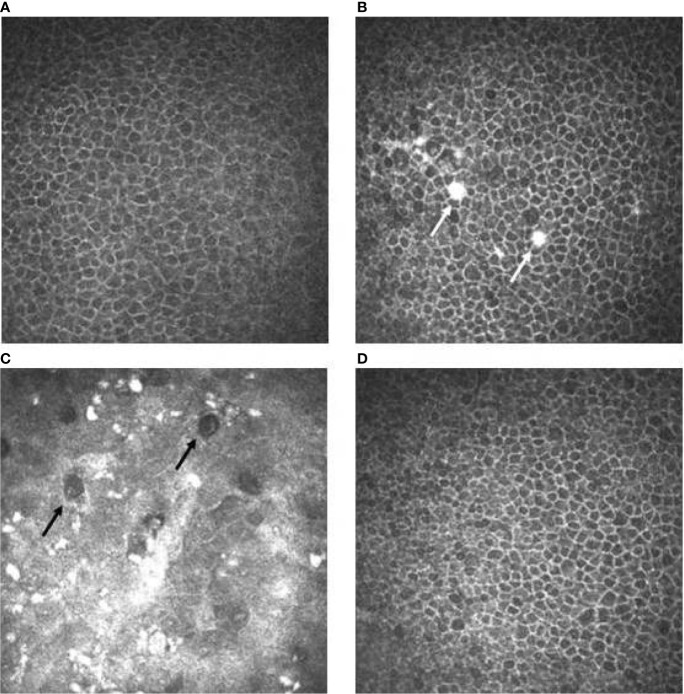
Confocal microscopy imaging revealing cornel toxicity secondary to ABT-414. **(A)** Normal basal epithelium before ABT-414 infusion. **(B)** Fifteen days after ABT-414 infusion note the appearance of epithelial hyperreflective white round spots (white arrows). A diffuse and mild increase of cells reflectivity is also present. **(C)** At 3 months follow-up, note the diffuse background of increased reflectivity, the increased number of the hyperreflective white round spots and the appearance of round hypo-reflective cysts (black arrows). **(D)** Twelve weeks after treatment discontinuation basal epithelial layers are normal.

CCM examination performed in the active treatment phase documented the appearance of white round spots located in the basal epithelial layers in all examined eyes, starting after a median of 1 drug infusion (range 1–3) (**Figure 3**).

The appearance of the round, epithelial hypo-reflective cystic structures was documented in all eyes (**Figure 3**), starting after a median of 2 drug infusion (range 1–4). The maximal severity of this side effect was graded as mild in 9 eyes (30%), moderate in 10 eyes (33%) and severe in 11 eyes (37%).

**Figure 4:**
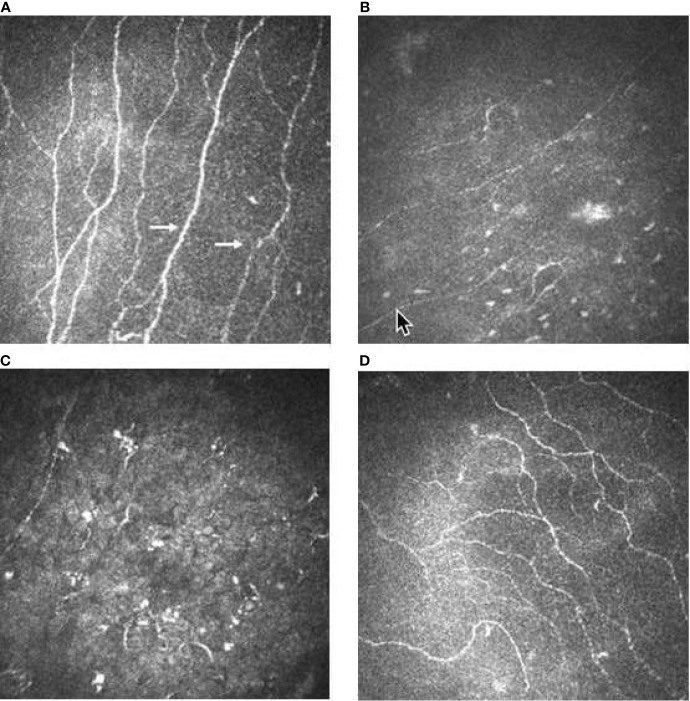
Confocal microscopy imaging revealing cornel toxicity secondary to ABT-414. **(A)** Normal subbasal nerve fibers (arrows) before ABT-414 infusion l. **(B)** Fifteen days after ABT-414 infusion note the initial fragmentation of the subbasal nerve fibers, **(C)** followed by its disappearance. **(D)** Twelve weeks after treatment discontinuation subbasal nerve fibers appeares normal.

The subbasal nerve plexus layer was characterized by fibers fragmentation and reduction starting at week 2, after a median of 1 drug infusion (range 1–3). Subsequently, the subbasal nerve plexus layer was then characterized by an evident disappearance in 22 eyes (73%) at week 4 and in all eyes (100%) at week 8 (**Figure 4**).

**Figure 5:**
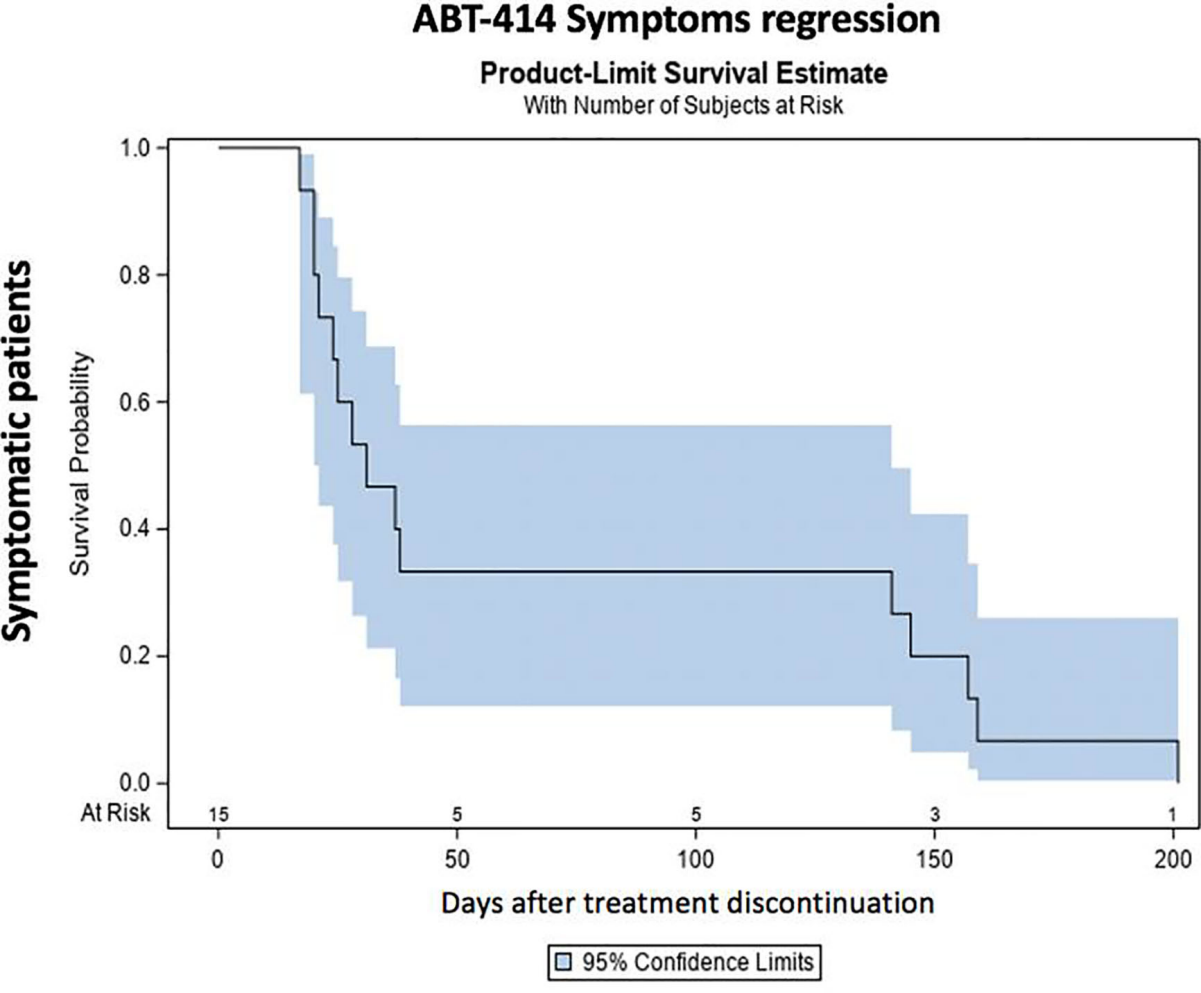
ABT-414 symptoms regression. Product-Limit Survival Estimate with number of subjects at risk.

**Figure 6:**
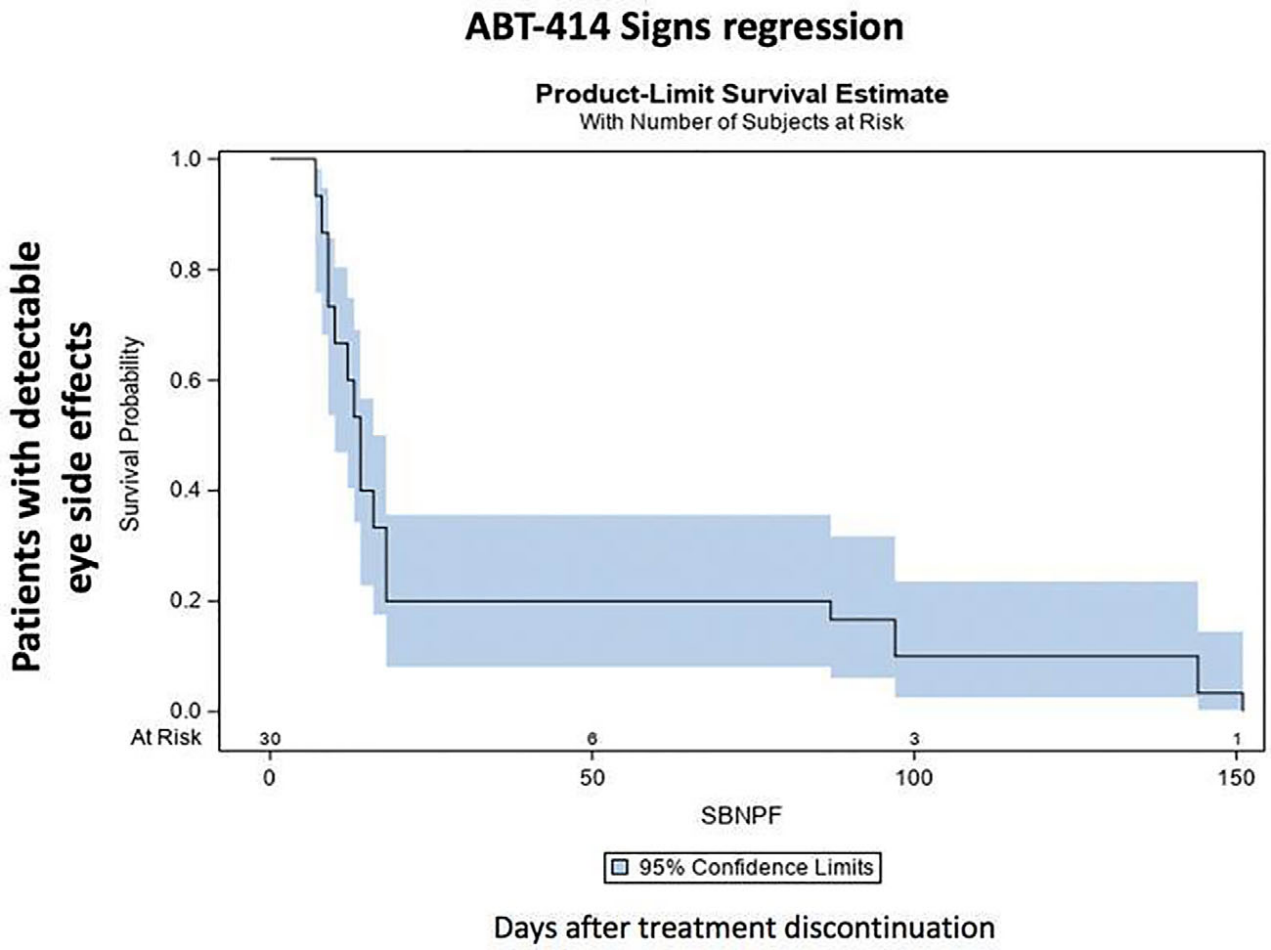
ABT-414 clinically detectable eye side effects regression. Product-Limit Survival Estimate with number of subjects at risk.

After drug discontinuation, signs and symptoms gradually attenuated. The prevalence and the median time of resolution of each specific ocular sign and symptom is reported on **Table 3**. Thirty days after drug discontinuation specific symptoms were present in 11 patients (73%). Symptoms rate reduced at 90 days (4 patients; 57%) and after 150 days (2 patients; 33%). After 210 days a single patient (25%) was characterized by a grade 1 photophobia, without other symptoms. The median time of symptoms resolution ranged from 38 days (eye pain) to 53 days (photophobia) (**Table 3**) (**Figure 5**) Thirty days after drug discontinuation specific clinical signs were present in 24 eyes (80%), including conjunctivitis (24 eyes; 80%) and keratitis (24 eyes; 80%). The prevalence of these signs reduced at 90 days (6 eyes; 42%) and after 150 days (4 eyes, 33%). After 210 days a single patient (25%) was characterized by a mild conjunctivitis and keratitis. The median time of signs resolution ranges from 14 days (corneal ulcer) to 38 days (superficial punctate epitheliopathy, corneal stroma edema, and intraepithelial cysts) (**Table 3**) (**Figure 6**).

One month after drug discontinuation CCM documented the persistence of specific corneal alterations in all eyes (100%), including basal epithelial hyperreflective white round spots (30 eyes, 100%), round cystic structure characterized by an hyperreflective wall (30 eyes, 100%) and fragmentation and depletion of sub-basal nerve plexus fibers (30 eyes, 100%). The prevalence of these alterations reduced at 90 days (6 eyes; 37%) and at 150 days (4 eyes; 33%), completely disappearing after 210 days. The median time of CCM signs resolution was 40 days. The prevalence and the mean time of resolution of each specific CCM sign is reported on **Table 3**. Clinically significant BCVA reduction was documented during treatment and at treatment discontinuation (65 ± 9 ETDRS score). This reduction persisted at 30 days follow-up (64 ± 11 ETDRS score), with a subsequent progressive restoration at 90, 150, and 210 days (70 ± 9; 77 ± 4; 84 ± 4 ETDRS score, respectively).

## Discussion

The first phase II randomized controlled study (NTELLANCE 2; NCT02343406) on ABT-414 alone or in combination with temozolomide in the treatment of EGFR amplified GBM was recently published (3). Two hundred sixty patients were randomized, and in the primary efficacy analysis the hazard ratio (HR) for the combination arm compared with the control arm was 0.71 (P = 0.062). Conversely, the efficacy of ABT-414 monotherapy was comparable to that of the control arm (HR = 1.04; P = 0.83). Therefore, the results of this trial suggested a possible role for the use of ABT-414 in combination with temozolomide in EGFR amplified recurrent GBM, especially in patients relapsing after completing 6 cycles of maintenance temozolomide. Unfortunately, the most frequent toxicity in ABT-414 treated patients was described as a reversible corneal epitheliopathy, occurring as grades 3–4 adverse event in 25%–30% of patients. Although in only few patients this side effect caused treatment discontinuation, the authors reported that the required dose reductions secondary to these side effects may have impacted the outcome of ABT-414 treatment. Therefore, a detailed analysis of this side effects appears mandatory. Different authors have reported ocular side effects secondary to the use of different EGFR inhibitors. Tullo et al. reported the development of conjunctival hyperemia, punctate keratopathy, blepharoconjunctivitis, and meibomitis during treatment with Gefitinib, an EGFR tyrosine kinase inhibitor (13). Ahn et al. reported the appearance of vortex keratopathy secondary to Vandetanib (an inhibitor of both EGFR and VEGFR2) (14). Other authors analyzed the incidence of ocular toxicity specifically related to the systemic treatment with ABT-414, reporting the common appearance of photophobia, keratoconjunctivitis sicca, blurred vision, and pain (10, 19). The phase I multicenter trial on the safety and efficacy of ABT-414 + temozolomide in patients with EGFR-amplified recurrent GBM (NCT01800695) reported that 87% of patients experienced any grade of ocular adverse events (2). Unfortunately, these side effects were not fully described and clinically characterized.

Focusing on the detailed clinical characterization of these side effects, our group have recently reported, for the first time, the CCM analysis of corneal side effects secondary to the use of ABT-414 in a cohort of patients affected by EGFR-amplified recurrent GBM (15). A total of 10 patients were consecutively enrolled with a median follow-up of 5 months, with no grade 4 toxicity and two (20%) grade 3 toxicities. CCM examination revealed in all eyes multiple and diffuse hyperreflective white round spots in the corneal basal epithelial layers (100%), progressive sub-basal nerve plexus layer fibers fragmentation (100%), and appearance of round cystic structures in the corneal epithelium (100%). Therefore, our group has demonstrated that ABT-414 toxicity is not only directed to the corneal epithelium, but also to corneal nerves, and that these side effects are detectable in all patients using CCM, an *in vivo* clinical tool allowing the study of corneal cellular structure at high magnification. Moreover, all these side effects reached the peak of prevalence and severity after a median of 2–3 infusion. After treatment discontinuation a significant trend toward reversibility was also documented, with a progressive corneal epithelium and sub-basal nerve plexus regeneration. However, in that pilot study a precise definition of a median time to resolution was not possible because of the limited follow-up (15).

In the present study we have confirmed that ocular side effects affect all treated patients and that ocular symptoms are experienced gradually by all patients during treatment. Moreover, in the present study, we have provided the median time of signs and symptoms resolution, ranging from 24 to 53 days. These data suggest that in a consistent proportion of patients signs and symptoms have a relatively long duration, probably impacting patients’ quality of life. The prolonged persistence of corneal side effects was also confirmed by CCM and, using this approach, we have demonstrated the parallel trend between symptoms and CCM signs, confirming the added value of using CCM when quantifying corneal side effects of affected patients.

The main limitation of this paper is probably the relatively limited number of patients enrolled. However, this limited court allowed us to fully investigate the corneal side effects secondary to ABT by a multimodal diagnostic approach, not limiting our investigation to macroscopic corneal changes, but also looking at a microscopic level.

In our study two patients reduced ABT dose to 1mg/Kg because of grade 2–3 side effects. This small number of patients did not allow us to evaluate the effect of dose reduction in ocular side effects. However, in future studies, it will certainly be interesting to understand whether a dose reduction can reduce the severity of ocular side effects.

Different pathogenetic hypothesis may explain corneal toxicity by ABT-414. The first hypothesis is that ABT-414 binding the EGFR in the corneal basal epithelial layer could release its cytotoxic effect into the cornea, mainly affecting the corneal epithelium and the sub-basal nerve plexus (9, 15). A second possibility is that ABT-414 could hypothetically cause ocular toxicity by an off-target mechanism: it is supposed the existence of a direct uptake of the unconjugated cytotoxin by tissue having a specific tropism for it (10, 19, 20). Another off-target mechanism suggests the existence of a direct uptake of the toxin in the lacrimal gland, whit subsequent secretion into the tear film (10, 19, 20).

In conclusion, corneal side effects secondary to ABT-414 are gradually experienced by all patients during treatment and are characterized by a relative prolonged persistence after treatment discontinuation.

## Conclusions

Corneal side effects by ABT-414 are detectable in all treated patients by CCM. Symptoms are experienced gradually by all patients during treatment, and the median time of symptoms resolution after treatment discontinuation ranges from 38 to 53 days. Corneal side effects by ABT-414 are reversible, and no grade 4 toxicity was documented. Nevertheless, given the high incidence of these side effects, the presence of 20%–30% of grade 3 toxicity and the relative prolonged persistence of symptoms, a study investigating the impact of ABT-414 on patients quality of life could help select those patients who could really benefit from this treatment.

## Data Availability Statement

The raw data supporting the conclusions of this article will be made available by the authors, without undue reservation.

## Ethics Statement

The studies involving human participants were reviewed and approved by Institutional Review Board (IRB) of the Veneto Institute of Oncology IOV-IRCCS (No. 08.2019). The patients/participants provided their written informed consent to participate in this study. Written informed consent was obtained from the individual(s) for the publication of any potentially identifiable images or data included in this article.

## Author Contributions

Conceptualization: RP, GL, EM, and LF. Methodology: RP, GL, EM, DL, GMa, GMi, VZ, and LF. Validation: RP and EM. Formal analysis: RP, DL, MP, GMa, and GMi. Investigation: RP, GL, EM, DL, GMa, GMi, MP, VZ, and LF. Resources: EM. Data curation: RP, GL, EM, DL, GMa, GMi, MP, VZ, and LF. Writing—original draft preparation: RP, DL, and LF. Writing—review and editing: RP, EM, and GL. Supervision: EM and RP. All authors contributed to the article and approved the submitted version.

## Conflict of Interest

The authors declare that the research was conducted in the absence of any commercial or financial relationships that could be construed as a potential conflict of interest.
